# Tolerogenic Lipid Nanoparticles for Delivering Self-Antigen mRNA for the Treatment of Experimental Autoimmune Encephalomyelitis

**DOI:** 10.3390/ph16091270

**Published:** 2023-09-07

**Authors:** Masaki Gomi, Yuka Nakayama, Yu Sakurai, Ryotaro Oyama, Koki Iwasaki, Mizuki Doi, Yi Liu, Mizuho Hori, Himeka Watanabe, Kohei Hashimoto, Hiroki Tanaka, Kota Tange, Yuta Nakai, Hidetaka Akita

**Affiliations:** 1 Laboratory of DDS Design and Drug Disposition, Graduate School of Pharmaceutical Sciences, Chiba University, 1-8-1, Inohana, Chuo-ku, Chiba 260-0856, Japan; masa0929.sprinter@gmail.com (M.G.); oyama.ryotaro.p3@dc.tohoku.ac.jp (R.O.); 1998.iwakou.22@gmail.com (K.I.); yama.md041@gmail.com (M.D.); yi.liu.s3@dc.tohoku.ac.jp (Y.L.); hiroki.tanaka.e1@tohoku.ac.jp (H.T.); 2 Laboratory of DDS Design and Drug Disposition, Graduate School of Pharmaceutical Sciences, Tohoku University, 6-3, Aoba, Aramaki, Aoba-ku, Sendai 980-8578, Japan; nakayama.yuka.r2@dc.tohoku.ac.jp (Y.N.); serendipity100111@gmail.com (Y.S.); hori.mizuho.t7@dc.tohoku.ac.jp (M.H.); himeka.watanabe.q7@dc.tohoku.ac.jp (H.W.); kohei.hashimoto.t7@dc.tohoku.ac.jp (K.H.); 3 Life Science Research Laboratory, NOF CORPORATION, 3-3, Chidoricho, Kawasaki-ku, Kawasaki 210-0865, Japan; kota_tange@nof.co.jp (K.T.); yuta_nakai@nof.co.jp (Y.N.)

**Keywords:** lipid nanoparticles, mRNA delivery, phosphatidylserine, antigen-specific tolerance

## Abstract

Multiple sclerosis is a disease caused by autoantigen-responsive immune cells that disrupt the myelin in the central nervous system (CNS). Although immunosuppressive drugs are used to suppress symptoms, no definitive therapy exists. As in the experimental autoimmune encephalitis (EAE) model of multiple sclerosis, a partial sequence of the myelin oligodendrocyte glycoprotein (MOG_35–55_) was identified as a causative autoantigen. This suggests that the induction of immune tolerance that is specific to MOG_35–55_ would be a fundamental treatment for EAE. We previously reported that lipid nanoparticles (LNPs) containing an anionic phospholipid, phosphatidylserine (PS), in their lipid composition, can be used to deliver mRNA and that this leads to proteins of interest to be expressed in the spleen. In addition to the targeting capability of PS, PS molecules avoid activating the immune system. Physiologically, the recognition of PS on apoptotic cells suppresses immune activation against these cells by releasing cytokines, such as interleukin-10 (IL-10) and transforming growth factor (TGF)-β that negatively regulate immunity. In this study, we tested whether mRNA delivery of autoantigens to the spleen by PS-LNPs causes the expression of MOG_35–55_ antigens with minimal immune stimulation and whether this could be used to treat an EAE model by inducing immune tolerance.

## 1. Introduction

Autoimmune diseases, of which more than 100 types have been identified, develop due to dysregulated adaptive immunity against self-antigens. Although the current therapeutics for the treatment of autoimmune diseases, such as steroids and other immunosuppressants, have successfully controlled their progression and relapse, these treatments potentially cause adverse, life-threatening effects resulting from nonspecific immunosuppression [1]. Thus, therapeutic approaches designed to repress self-reactive immune responses are needed.

The delivery of self-antigens with minimal immune-stimulation is emerging as a promising strategy for inducing antigen-specific tolerance [2]. In contrast to proinflammatory conditions, in which antigen-presenting cells (APCs) acquire both antigens and stimulatory signals to activate adaptive immunity, the non-inflammatory state causes APCs to induce immunotolerance to the antigens in APC [3]. Various studies dealing with the delivery of self-antigens to induce disease-specific tolerance using nanocarriers as the use of these carriers indicate that this approach has an advantage in that it enhances the uptake of antigens by APCs [4]. However, nanocarriers themselves can also potentially induce immuno-stimulation [5,6]. Thus, the design of a material that avoids immune-stimulation or the co-delivery of immunosuppressive drugs represents an immune-tolerogenic approach to this issue [7,8].

In the present study, we report on a successful induction of immune tolerance with the delivery of autoantigen-encoding mRNA by spleen-targeted lipid nanoparticles (LNPs) using an anionic phospholipid phosphatidylserine (PS). Two potential advantages are expected as the result of the incorporation of PS in the lipid composition of the LNP: macrophage-targeting and immune-suppressive activity. Our previous comprehensive analysis of the relationship between the physicochemical properties of LNPs and their accumulation in lymph nodes after the s.c. administration revealed that the entrapment of these particles by the macrophage is a crucial driving force for their extensive accumulation in the lymphatic system [9,10]. We recently reported on the development of an LNP that efficiently delivers the mRNA to the spleen with the aid of a PS-derivate designed to be soluble in alcohol [11]. Intravenously administered PS-LNP delivered mRNA to spleen-resident macrophages via PS-receptor (i.e., Tim-4), expressed on macrophages [11]. PS has a physiological role in immunotolerance, called an “Eat-me” signal [12]: the phagocytes recognize apoptotic cells through PS exposed on the outer leaflets of the cell membrane, and this subsequently triggers immunosuppression [13]. The engulfment of PS-exposing cells accelerates the release of immunosuppressive cytokines such as TGF-β and IL-10 [14]. In addition, the disruption of PS signaling leads to autoimmune responses. Therefore, PS in apoptotic cells plays a key role in homeostasis of the immunological condition [15,16]. Thus, we hypothesized that our PS-incorporated LNP (PS-LNP) could be used to deliver mRNA-encoded self-antigens to APCs without immune-stimulation and that this would lead to the development of an anti-inflammatory environment. This property would eventually induce antigen-specific immune-tolerance. Indeed, several studies have demonstrated that PS-loaded nanomaterials suppressed immune responses to the incorporated proteins or peptides [17,18,19]. Also, a previous study reported on the therapeutic potential of delivering self-antigen mRNA using an anionic lipoplex as an mRNA carrier [20].

Here, we report on a therapeutic effect against experimental autoimmune encephalomyelitis (EAE), the most common animal model of multiple sclerosis, based on the delivery of the myelin oligodendrocyte protein including 35–55 (MOG_35–55_)-expressing mRNA as a pathogenic epitope.

## 2. Results

### 2.1. The Immune-Suppressive Effect of the PS-LNP

We prepared PS-LNPs with the formulation that had been optimized in a previous report (COATSOME^®^ SS-OP/cholesterol/Dilinoleoyl-sn-glycero-3-phosphoserine = 52.5/40/7.5) [11]. Since we prepared the mRNA-LNPs using a microfluidic device that was different from that used in our previous study, we assessed the ability of the present PS-LNPs to target the spleen after intravenous injection. We evaluated the accumulation of PS-LNP and the functional delivery of mRNA to the spleen using the Luciferase reporter as a reporter gene. As a comparison, PC-LNPs that contained 1,2-Dioleoyl-sn-glycero-3-phosphocholine (DOPC) instead of 18:2 PS were injected. The diameters, mRNA recovery ratios (RR) and encapsulation efficiencies (EE) of PS-LNP and PC-LNP were comparable (Table 1). While the zeta-potential of PC-LNP was nearly neutral, the PS-LNPs exhibited a negative charge due to presence of serine and phosphate residues (Table 1). The PS-LNP encapsulating Luciferase (Luc)-mRNA (PS-LNP_Luc_) accumulated more efficiently and delivered the mRNA cargo to the spleen better compared to the control PC-LNP. These data indicate that the PS-LNP exhibits a spleen-targeting ability regardless of the device used to prepare it (Figure S1). Moreover, the physicochemical properties of the current LNPs and the previously reported LNP were almost the same (Table S1).

**Figure 1:**
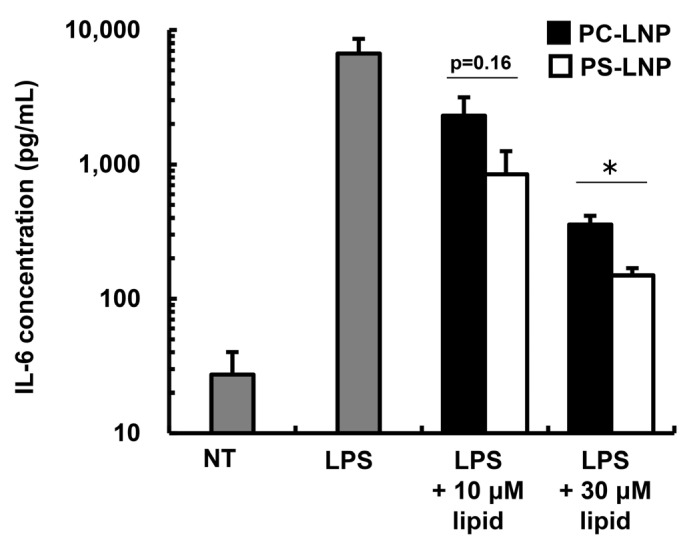
Suppressive effect of PS-LNP on inflammatory cytokine production from macrophages. Raw cells were incubated with 100 ng/mL of lipopolysaccharide (LPS) in the presence or absence of PC-LNP or PS-LNP encapsulating Luc-mRNA, and then IL-6 concentration was evaluated 24 h after the addition of LPS and LNPs. *: *p* < 0.05 (Student’s *t*-test). Data represent the mean with standard error (S.E.).

We then assessed the immune-suppressive effect of the PS-LNPs. Inflammatory cytokine interleukin(IL)-6 from macrophage-derived cells stimulated by lipopolysaccharide (LPS) was regarded as an indicator of PS-inducing immunosuppression in previous reports [21,22,23]. A murine macrophage cell line, Raw264.7 cells, was incubated with the LPS and/or the PS-LNP, and the amount of cumulative IL-6 concentration was then measured 24 h after the addition (Figure 1). As a comparison, PC-LNP was applied to the Raw264.7 cells. Both LNPs suppressed IL-6 production through LPS stimulation, but the IL-6 concentration in the PS-LNP treated group was significantly lower than that for the PC-LNP. In addition, we observed no toxicity by PS- and PC-LNPs within the indicated lipid concentration (Figure S2). Moreover, neither PS- nor PC-LNP induced IL-6 production in the absence of LPS (Figure S3).

### 2.2. PS-LNP Loaded with MOG_27–63_ mRNA Ameliorates EAE

**Figure 2:**
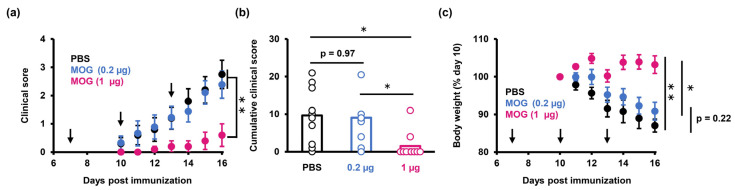
PS-LNP loaded with MOG_27–63_ mRNA ameliorates EAE. (**a**) Clinical scores in EAE mice treated with MOG_27–63_ mRNA (0.2 µg or 1 µg) or PBS. (**b**) Cumulative clinical scores which are calculated from (**a**). One-way ANOVA followed by Tukey’s HSD test was performed. *: *p* < 0.05, **: *p* < 0.01. (**c**) Weight changes in EAE mice. (**a**,**c**) Data represent the mean with S.E. Arrowheads represent the days on which the mice were treated.

To test the therapeutic potential of PS-LNP in EAE, we treated MOG_35–55_-immunized mice with PS-LNPs that were loaded with MOG_27–63_ mRNA (PS-LNP_MOG_) at two different doses (0.2 µg or 1 µg mRNA per mouse) at 7, 10 and 13 days after the immunization. The results showed that 1 µg mRNA was sufficient to suppress both the severity of the EAE clinical scores and the decrease in body weight, whereas 0.2 µg mRNA failed to exert a measurable effect on EAE (Figure 2).

**Figure 3:**
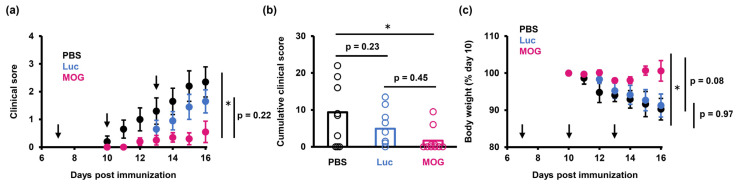
PS-LNP needs encapsulation of MOG_27–63_ mRNA to ameliorate EAE. (**a**) Clinical scores in EAE mice treated with MOG_27–63_ mRNA (1 µg), luciferase mRNA (1 µg), or PBS. (**b**) Cumulative clinical scores are calculated from (**a**). One-way ANOVA followed by Tukey’s HSD test was performed. *: *p* < 0.05. (**c**) Weight changes in EAE mice. (**a**,**c**) Data represent the mean ± S.E. Arrowheads represent the days the mice were treated.

To prove antigen specific-immunotolerance, we next compared the therapeutic effects of PS-LNPs loaded with a different disease-irrelevant protein, Luc. As a result, PS-LNP_Luc_ failed to significantly inhibit the onset of EAE, indicating that the expression of the MOG_35–55_ sequence is essential for inducing tolerance in EAE (Figure 3). On the other hand, the EAE mice treated with the PS-LNP_Luc_ exhibited lower clinical scores compared to the PBS-treated mice, but this difference was not significant.

### 2.3. Evaluation of Infiltrating Immune Cells in the Central Nervous System

**Figure 4:**
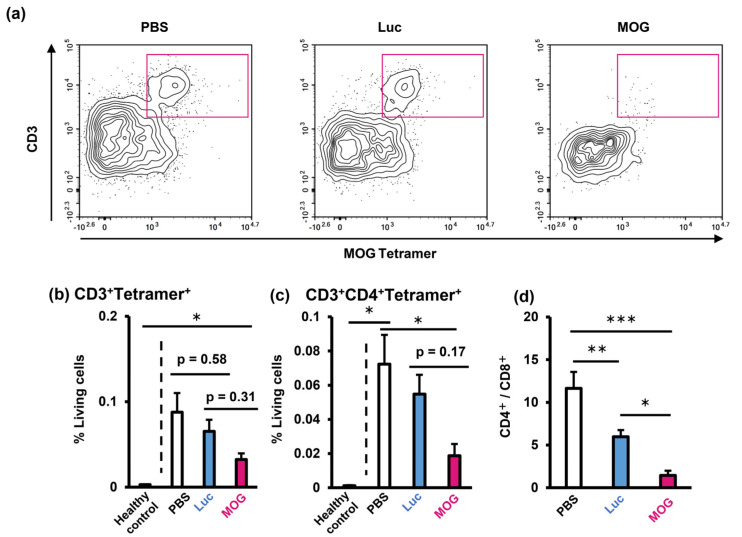
Treatment with PS-LNP suppresses infiltration of abnormal T cells into the brain. (**a**) Representative plots of brain-infiltrating immune cells (CD45^+^). Magenta squares indicate MOG_35–55_-reactive T cells. (**b**) Infiltration of MOG_35–55_-reactive T cells (CD45^+^/CD3^+^Tetramer^+^) and (**c**) CD4^+^ T cells (CD45^+^/CD3^+^/CD4^+^/Tetramer^+^) into the brain. (**d**) CD4^+^/CD8^+^ ratio of brain-infiltrating T cells. (b, c) Data represent the mean with S.E. One-way ANOVA followed by Tukey’s HSD test was performed. *: *p* < 0.05; **: *p* < 0.01; ***: *p* < 0.001.

Abnormal infiltration of autoreactive T cells, especially CD4^+^ cells, into the central nervous system is a common feature in EAE [24]. Thus, we investigated T-cell infiltration into the brain of EAE mice that had been treated with PS-LNP to evaluate its effect on pathogenic immune cell recruitment. We combined a MOG_35–55_/I-Ab tetramer with surface staining to detect MOG_35–55_-reactive T cells. Treatment with the PS-LNP_MOG_ reduced MOG_35–55_-reactive T cells in the brains of EAE mice (Figure 4a,b). PS-LNP_MOG_ significantly reduced CD3^+^CD4^+^Tetramer^+^ cells compared to PBS (Figure 4b). The infiltration of both CD4^+^ cells were suppressed by PS-LNP_MOG_ (Figure 4c). In addition, the CD4^+^/CD8^+^ ratio of the brain-infiltrating T cells was strikingly decreased in the PS-LNP-treated mice (Figure 4d). Moreover, PS-LNP_MOG_ more meaningfully reduced the CD4^+^/CD8^+^ ratio than the PS-LNP_Luc_.

### 2.4. Evaluation of Immune Status in the Spleen

**Figure 5:**
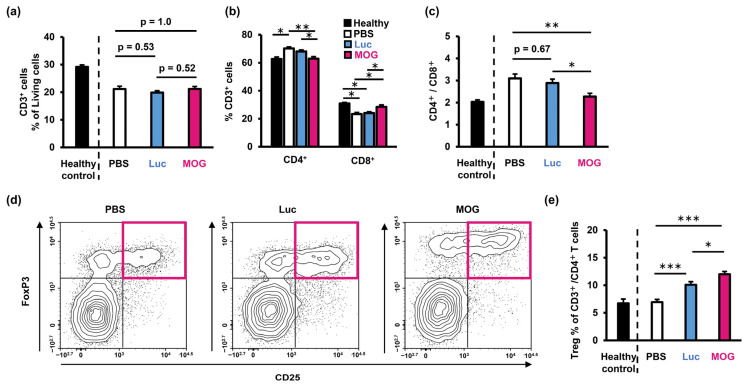
Treatment with PS-LNP modulates splenic T cell properties. (**a**) Frequency of total T cells and (**b**) CD4^+^ and CD8^+^ T cells in splenocytes from EAE mice. (**c**) CD4^+/^CD8^+^ ratio of the splenic T cells. (**d**) Representative plots of CD4+ T cells. Magenta squares indicate Tregs. (**e**) Frequency of Tregs in the splenic CD4^+^ T cells. (**a**–**c**,**e**) Data represent the mean with S.E. One-way ANOVA followed by Tukey’s HSD test was performed. *: *p* < 0.05; **: *p* < 0.01; ***: *p* < 0.001.

We next analyzed T cells in the spleen, which is the target tissue of the PS-LNPs, to study the direct effects of the PS-LNP on the splenic microenvironment. While the total splenic T-cell frequency was comparable between the PBS-treated mice and the PS-LNP-treated mice (Figure 5a), the proportions of both CD4^+^ cells and CD8^+^ cells were normalized by PS-LNP treatment (Figure 5b). Furthermore, the CD4^+^/CD8^+^ ratio in the mice that had been treated with PS-LNP_MOG_ was decreased compared to that for the PBS-treated mice (Figure 5c). Furthermore, the CD4^+^/CD8^+^ ratio of the PS-LNP_MOG_-treated mice was comparable with the healthy control, indicating the CD4^+^/CD8^+^ balance was normalized. The PS-LNP treatment also increased the frequency of regulatory T cells (Treg: CD3^+^ CD4^+^ CD25^+^ FoxP3^+^), and PS-LNP_MOG_ resulted in the further expansion of Treg (Figure 5d,e).

To evaluate the immune responses to the MOG_35–55_ epitope, we restimulated splenocytes derived from the EAE mice that had been treated with MOG_35–55_ and measured cytokine production (Figure S4). The results confirmed that the unstimulated splenocytes produce negligible amounts of cytokines, indicating that cytokine production reflects MOG_35–55_-specific responses (Figure S4). The production of IL-2 decreased in the mice that had been treated with the PS-LNP regardless of the types of encapsulated mRNA. In addition, we observed a reduced production of IL-17A, a proinflammatory cytokine that plays a pivotal role in multiple sclerosis [25], only in the mice that had been treated with the PS-LNP_MOG_. IL-2, which is responsible for T-cell proliferation, was significantly reduced by both LNP_Luc_ and LNP_MOG_ injection, suggesting that PS itself can decrease cytokine production. PS-LNP more substantially decreased IL-4 and IL-10 production than PC-LNP. On the other hand, the production of IFN-γ, IL-6 and TNF were unchanged by the treatment with PS-LNPs. We found that the expression of the MOG_35–55_ epitope from PS-LNP_MOG_ induced MOG_35–55_-specific tolerance, and the formation of an immune-suppressive microenvironment by the PS-LNP synergistically enhanced this effect.

## 3. Discussion

We report herein on the LNP-based delivery of mRNA to induce antigen-specific tolerance as a therapeutic approach for the treatment of multiple sclerosis. mRNA is a desirable tool for introducing the production of various self-antigens for tolerance induction since it can theoretically induce specific tolerance to any antigen of interest by appropriately tailoring the mRNA sequences.

We incorporated PS into the LNP formulation inspired by the mechanisms of tolerogenic clearance of apoptotic cells through PS-mediated signaling. Indeed, the results demonstrated that the PS-LNP not only functioned as a carrier of self-antigen mRNA but also had some tolerogenic features. The PS-LNP suppressed in vitro IL-6 production in Raw264.7 cells in response to an LPS treatment, only at the highest concentration (Figure 1, 30 μM). In our in vivo study, 15 nmol of PS molecules were administered. Given that 10% of the once intravenously administered LNP at the same dosages (200 nmol lipid, 1 μg mRNA) that were used in the therapeutic experiment with the EAE model (Figure 2, Figure 3, Figure 4 and Figure 5) was accumulated and uniformly distributed in the spleen with a volume of 60 mm^3^ [26], the PS concentration in the spleen was estimated at 250 μM. Based on our result, this concentration is considered enough to suppress macrophage activation (Figure 1). We estimate that these immunosuppressive features of the PS-LNP may be advantageous for the induction of self-tolerance because the systemic injection of LNPs was found to activate antigen-specific immune responses in some cases [27,28].

The PC-LNP also caused a reduction in IL-6 production (Figure 1). This reduction may be explained, at least partially, by the capture of protons in endosomes by the ionizable lipid SS-OP. The SS-OP in the LNPs that were used contains tertiary amines in its structure that allow it to capture protons. Inhibiting endosome maturation by inhibiting acidification leads to the attenuation of inflammatory signaling pathways [29]. Indeed, in a previous study, we showed that LNPs containing structures that were similar to SS-OP suppress enteritis when administered to mouse models of colitis [30]. The accumulation of the PC-LNP in the spleen was nearly two times lower than that for the PS-LNP (Figure S1a,b). Since the reduction of cytokine production by LNPs is highly dependent on concentration (Figure 1), the inhibitory effect of PC-LNP on the inflammatory cytokine production in the spleen would be expected to be marginal.

One significance of the presented approach is the need for lower doses of mRNA. In a previous work using a lipoplex of mRNA, 20 µg mRNA was administered per mouse [17]. In comparison, the PS-LNP in the present study successfully inhibited EAE at a dose of just 1 µg mRNA. However, a slight suppression in clinical score progression and weight loss were observed in the case of the 0.2 ug mRNA dose (Figure 2). It is possible that the coexistence of a sufficient amount of PS molecules and antigen translated from mRNA is important in suppressing the progression of EAE pathology.

The amelioration of EAE was associated with an expansion of Treg in the spleen. PS-LNP efficiently delivers mRNA to splenic macrophages, especially red pulp macrophages and marginal zone-residential macrophages [11]. As recent studies revealed that marginal zone-residential macrophages are involved with physiological immune tolerance to apoptotic cells [31], we hypothesized that these cell subpopulations would render antigen-presentation of the MOG_35–55_ epitope in a tolerogenic manner, leading to Treg induction. Tregs not only suppress antigen-specific immune responses but also exert broadly tolerogenic functions that include the secretion of anti-inflammatory cytokines such as IL-10 and TGF-β [32]. Not only PS-LNP_MOG_ but also PS-LNP_Luc_ caused an increase in Treg numbers, possibly due to the function of PS itself (Figure 5e). It is possible that this noncognate suppression may also contribute to the partial therapeutic effects of PS-LNP loaded with disease-irrelevant mRNA (PS-LNP_Luc_ in Figure 4).

In addition, treatment with PS-LNP_MOG_ reduced the CD4^+/^CD8^+^ ratio of T cells in both the brain and the spleen (Figure 4d and Figure 5c). CD4^+^ T cells play a central role in the pathogenesis of multiple sclerosis [24,33]. On the other hand, the role of CD8^+^ T cells is unclear. In a previous report, the CD8^+^ cell depletion by antibodies was found to not significantly affect the clinical score, although CD4^+^ cell depletion completely inhibited disease progression in the EAE model [34]. In addition, Leuenberger et al. reported that the adoptive transfer of the MOG_35–55_ peptide-stimulated CD8^+^ T cells failed to induce EAE symptoms though CD8^+^ cells in CNS [35]. In addition, one study reported that CD8^+^ stimulated with the MOG_35–55_ peptide inhibited CD4^+^ T-cell priming by dendritic cells through the production of IL-10, thereby inhibiting disease progression [36]. In our results, although the PS-LNP_MOG_ significantly increased the level of CD8^+^ T cells in CNS (Figure 4d), this increase may impair the differentiation of CD4^+^ T cells in the therapeutic effect of EAE. Accordingly, the decreased predominance of the CD4^+^/CD8^+^ T cells ratio would be closely related to the protective effects of PS-LNP.

This study’s main limitation is not demonstrating the advantage of PS incorporation into lipid composition on the therapeutic effect against EAE and the therapeutic effect against more advanced stages of the EAE model. In the near future, further investigation should be required for an elucidation of the role of PS in LNP and its advantage against the natural helper lipid, PC, for evaluating the therapeutic effect of PS-LNP against after onset of symptoms.

## 4. Materials and Methods

### 4.1. Materials

COATSOME^®^ SS-OP was supplied by NOF CORPORATION (Tokyo, Japan), and 1,2-Dimyristoyl-rac-glycero-3-methylpolyoxyethylene-2000 (DMG-PEG2000), DOPC was purchased from NOF CORPORATION. Dilinoleoyl-PS was purchased from Avanti Polar Lipids. Cholesterol was purchased from Sigma-Aldrich (St. Louis, MO, USA). Phosphate-buffered saline (PBS) without Ca^2+^ and Mg^2+^, tert-Butanol, DL-malic acid, 2-(N-morpholino) ethanesulfonic acid (MES), RPMI-1640 medium with L-Glutamine and bovine serum albumin (BSA) were purchased from Nacalai Tesque (Kyoto, Japan). Amicon Ultra-15–100 K centrifugal units were purchased from Merck Millipore (Burlington, MA, USA). Antibodies are listed in Table S2.

### 4.2. Mice

Female C57BL6/J mice were purchased from Japan SLC, Inc. (Shizuoka, Japan). Prior to the studies, the experimental protocols were reviewed and approved by the Tohoku University Animal Care Committee (Approved No. 2021Yakudou-011-01).

### 4.3. Induction of EAE

In this study, 10-week-old C57BL6/J mice were acclimated to the environment for 7 days. According to the supplier’s protocol, the mice were immunized using a Hooke Kit™ MOG_35–55_/CFA Emulsion PTX (Hooke Laboratories, Lawrence, MA, USA). Briefly, 100 µL of MOG_35–55_/CFA emulsion was subcutaneously injected at the upper back and the lower back (day 0). In addition, 165 ng of pertussis toxin in the Hooke Kit™ was intraperitoneally injected on day 0 and 1. The partial sequence MOG_35–55_ peptide is known as an epitope on the MHC class II of C57BL/6 mice [37,38].

### 4.4. In Vitro Transcription of mRNA

Sequences corresponding to firefly luciferase (Luc) mRNAs or MOG_27–63_ mRNA were cloned into an in vitro transcription vector (pT7 vector for mRNA) (VectorBuilder Inc., Chicago, IL, USA). The sequence of the MOG_27–63_ mRNA was constructed, from which the MOG_35–55_ epitope was generated and presented on MHC II, as previously reported [20]. Following the supplier’s protocol, the mRNAs were prepared using the Takara IVTpro T7 mRNA Synthesis Kit (TaKaRa Bio Inc., Shiga, Japan). For the transcription procedure, 1-methylpseudouridine-5′-triphosphate (m1ΨTP) (TriLink Biotechnologies Inc., San Diego, CA, USA) was used instead of uridine-5′-triphosphate (UTP) to reduce the immunogenicity of the mRNAs [39]. Then, the 5′ end of the transcribed RNA was capped using a Vaccinia Capping Enzyme (TaKaRa), and then Cap0 was converted to Cap1 with a mRNA Cap 2′-O-Methyltransferase (TaKaRa). Contaminated dsRNA was removed with a cellulose column. All of the mRNA sequences are shown in the Supplementary experimental section.

### 4.5. Preparation of mRNA-Loaded LNPs

The mRNAs were dissolved in malic acid buffer (20 mM malic acid and 30 mM NaCl, pH3.0). The lipid mixture (SS-OP/18:2 PS/Cholesterol/DMG-PEG2000 = 52.5/7.5/40/1 [molar ratio]) was prepared in 90% tert-Butanol (tert-Butanol and ultrapure water were mixed at the volume ratio of 9:1). The malic buffer was used according to our previous optimization (unpublished data). Then, 6.3 µg/mL mRNA solution and 5 mM lipid solution were mixed using a microfluidic device which was graciously provided by Professor Manabu Tokeshi (Hokkaido University) (total flow rate, 1.0 mL/min; flow ratio, water/alcohol = 3/1 [*v*/*v*]) [40]. One mL of MES buffer was added to the LNP solution under vigorous mixing and then diluted up to 4 mL. The dilutant was ultrafiltrated using an Amicon Ultra-15 (MWCO 100 kDa). The concentrate was diluted by PBS up to 4 mL and then ultrafiltrated with the Amicon Ultra-15. The procedure was repeated twice. After the volume of the LNP solution was less than 250 μL, the volume of LNP solution was adjusted to 500 μL. The size, polydispersity index and zeta potential of the LNPs were measured with a ZetaSizer Nano ZS (Malvern Instruments Ltd., Malvern, UK). The recovery and encapsulation of the mRNAs were evaluated with the Quant-it TM RiboGreen RNA Assay Kit (Thermo Fisher Scientific Inc., Waltham, MA, USA). The fluorescent intensity of the LNP solution was measured in the presence of Quant-it TM RiboGreen RNA solution with a SpectraMax iD5 (Molecular Devices, San Jose, CA, USA). This fluorescent intensity indicates unencapsulated mRNA. To evaluate encapsulation efficiency, the fluorescent intensity of LNP solutions that were dispersed by 0.4% (*w*/*v*) triton X-100 was measured. This intensity means the entire mRNA. The recovery rate (%RR) was calculated from the fluorescent intensity in the presence of 0.4% triton X-100. The encapsulation efficiency (%EE) was calculated by subtracting the unencapsulated mRNA concentration from the entire mRNA concentration. As a control of anionic phospholipid PS, DOPC was incorporated into the lipid composition instead of 18:2 PS (PC-LNP).

### 4.6. Effect of PS-LNP on a Production of IL-6 from Activated Macrophages

Raw264.7 cells were obtained from ATCC. Raw264.7 cells were maintained in RPMI-1640 supplemented with 10% FBS, 100 IU/mL penicillin and 100 μg/mL streptomycin. The Raw264.7 (2.0 × 10^4^) cells were plated on a 24-well plate. At the same time, PS-LNP and PC-LNP were mixed with cell culture medium at 3, 10 and 30 μM as a lipid concentration. A 100 μg/mL solution of lipopolysaccharide (LPS, Sigma-Aldrich) was then added, and the supernatants were harvested 24 h after the LPS addition. The IL-6 concentrations in the supernatants were evaluated by means of a Mouse IL-6 Quantikine ELISA Kit | mIL-6 QKit (R&D systems, Minneapolis, MN, USA). The standard curve was fitted by using a 4-parameter logistic regression using R software (version 4.3.1).

### 4.7. LNP-Based Treatment of EAE Mice

Various doses of mRNA-loaded LNPs (0.2 µg or 1 µg mRNA per mouse) or PBS were intravenously injected to EAE mice via the tail vein on days 7, 10 and 13 after immunization. From day 10 after immunization, the mice were weighed daily and scored for clinical symptoms according to the following criteria: 0, no symptom; 1, limp tail; 2, partial hind limb paralysis; 3, complete hind limb paralysis; 3.5, complete hind limb paralysis and minimal moving; 4, partially fore limb paralysis; and 5, moribund or dead.

### 4.8. Cell Isolation from Mouse Tissues

The spleen and brain were harvested from EAE mice on day 16 after immunization. The harvested tissues were minced through a 70-μm cell strainer using the plunger of a 3-mL syringe (TERUMO, Tokyo, Japan) to produce a single-cell suspension. For preparing splenocytes, erythrocytes were removed through hypotonic lysis using RBC lysis buffer (Sigma Aldrich, St. Louis, MO, USA).

### 4.9. Flow Cytometry

Cells harvested from the spleen or brain were suspended in flow cytometry buffer (PBS supplemented with BSA [0.5 *w*/*v*%] and sodium azide [0.1 *w*/*v*%]). For blocking Fc receptors, 2 × 10^6^ cells were incubated with 10 µg/mL anti-CD16/32 antibody at 4 °C for 30 min. The cells were stained with fluorescence-conjugated antibodies at 4 °C for 30 min for surface staining. Dead cells were simultaneously stained by Fixable Viability Dye eFluor780 (eBioscience, San Diego, CA, USA). CD4^+^, CD8^+^ and T-reg cells were gated as shown in Supplemental Figures S2 and S3. For intracellular staining, the cells were fixed and permeabilized using the Transcription Factor Buffer Set (BD Biosciences, San Jose, CA, USA) followed by incubated with an anti-FoxP3 antibody. When we analyzed MOG_35–55_-specific CD4+ T cells, tetramer staining was performed using T-Select I-Ab MOG_35–55_ Tetramer-PE (MBL Life Science, Tokyo, Japan) before surface staining at room temperature for 30 min. The cells were analyzed using NovoCyte (Agilent, Santa Clara, CA, USA). Brain cells were gated according to Figure S5. Splenocytes were gated in accordance with Figure S6.

### 4.10. Ex Vivo Stimulation of Splenocytes

To assess antigen-specific immunoreaction, 4 × 10^5^ splenocytes from EAE mice were plated on a 96-well plate and stimulated for 72 h with 200 µL of 100 µg/mL MOG_35–55_ in RPMI-1640 medium supplemented with FBS (1 *v*/*v*%), penicillin ([100 U/mL] and streptomycin (100 mg/mL). The supernatant was analyzed for cytokine production using a BD CBA Mouse Th1/Th2/Th17 Cytokine Kit (BD Bioscience) following the supplier’s protocol. The data were processed using CBA Analysis Software v3.0 (BD Bioscience).

### 4.11. Statistical Analysis

For pair-wise comparison, Student’s *t*-test was performed. For a comparison of more than three groups, ANOVA followed by Tukey’s HSD test was performed. If the *p*-value was less than 0.05, the difference was regarded as statistically significant.

## 5. Conclusions

We herein presented a promising approach for treating autoimmune diseases using mRNA-LNPs to deliver self-antigen mRNA using an EAE model. The study showed that PS-LNP encapsulating mRNA encoding the MOG_35–55_ peptide can reduce EAE-associated symptoms. Moreover, in the brain, PS-LNP induces Treg cells, which are crucial in suppressing auto-immunity. In the spleen, PS-LNP can recover the CD4^+^/CD8^+^ cell balance. This approach can reduce side effects and improve patient outcomes compared to current treatments for autoimmune diseases, which often involve broad immunosuppression. Overall, this study provides evidence for the potential use of tolerogenic PS-LNP as a targeted and selective approach for treating autoimmune diseases.

## 6. Patents

H.T., Y.N. (Yuta Nakai), K.T., and H.A. are the inventors of a patent pending (WO2013/073480 and WO2016/121942) on COATSOME SS-OP.

## Figures and Tables

**Table 1:** The characterization of the LNPs.

Sample	Z-Average (nm)	PdI	Zeta-Potential (mV)	RR (%)	EE (%)
PC-LNP_Luc_	126.7 ± 2.3	0.14 ± 0.06	−2.1 ± 2.0	80.6 ± 17.5	71.4 ± 9.6
PS-LNP_Luc_	131.4 ± 5.2	0.12 ± 0.02	−18.7 ± 1.3	82.1 ± 9.8	61.7 ± 3.3
PS-LNP_MOG_	133.1 ± 0.9	0.15 ± 0.04	−21.2 ± 2.3	94.7 ± 6.5	40.8 ± 4.3

PdI: Polydispersity Index, RR: Recovery ratio of mRNA, EE: Encapsulation efficiency of mRNA.

## Data Availability

Data is contained within the article and supplementary material.

## Supplementary Materials

The following supporting information can be downloaded at: https://www.mdpi.com/article/10.3390/ph16091270/s1, Figure S1: Biodistribution and mRNA delivery of intravenously injected LNPs; Figure S2: The effect of PS- and PC-LNP on cell viability; Figure S3: IL-6 production by PS- or PC-LNP in the absence of LPS; Figure S4: MOG_35–55_-responsive cytokine production by splenocytes; Figure S5: Gating strategy of cells from brain; Figure S6: Gating strategy of splenocytes; Table S1: The characterization of LNPs prepared by iLiNP and Nanoassemblr; Table S2: Antibodies used for flow cytometry. Ref. [41] is cited in the supplementary materials.
