# Supplementary material for: Tolerogenic Lipid Nanoparticles for Delivering Self-Antigen mRNA for the Treatment of Experimental Autoimmune Encephalomyelitis
Source: Pharmaceuticals (Basel). 2023 Sep 7;16(9):1270. doi: 10.3390/ph16091270 (PMC10537621; doi:10.3390/ph16091270)
Supplement: Supplementary file 1 [file pharmaceuticals-16-01270-s001.zip › pharmaceuticals-2574646-supplementary.pdf]

Supporting Information

Tolerogenic Lipid Nanoparticles for Delivering Self-antigen mRNA for the Treatment of Experimental Autoimmune Encephalomyelitis

Masaki Gomi [1,2], Yuka Nakayama [2], Yu Sakurai [2], Ryotaro Oyama [1,2], Koki Iwasaki [1], Mizuki Doi [1,2], Yi Liu [1,2], Mizuho Hori [2], Himeka Watanabe [2], Kohei Hashimoto [2], Hiroki Tanaka [1], Kota Tange [3], Yuta Nakai [3], and Hidetaka Akita [2,*]

1. Laboratory of DDS Design and Drug Disposition, Graduate School of Pharmaceutical Sciences, Chiba University, 1-8-1, Inohana, Chuo-ku, Chiba, Chiba, 260-0856 Japan

2. Laboratory of DDS Design and Drug Disposition, Graduate School of Pharmaceutical Sciences, Tohoku University, 6-3, Aoba, Aramaki, Aoba-ku, Sendai, Miyagi, 980-8578 Japan

3. Life Science Research Laboratory, NOF CORPORATION, 3-3, Chidoricho, Kawasaki-ku, Kawasaki, Kanagawa, 210-0865 Japan

* Corresponding author: hidetaka.akita.a4@tohoku.ac.jp

Supplementary experimental section

In vivo imaging of LNP biodistribution and functional mRNA delivery

DiR (0.2 mol%)-labeled LNPs loaded with luciferase mRNA were intravenously injected into mice via the tail vein (1 µg mRNA / 200 µL PBS). The mice were intraperitoneally injected with 3 mg of luciferin potassium in 200 µL PBS at 6 h after the LNP injection. At 5 min after the luciferin injection, the mice were euthanized, and the spleens and livers were harvested. The biodistribution of the LNPs and luciferase activity were then evaluated by IVIS Lumina II (PerkinElmer, Waltham, MA, USA).

Template DNA sequences for Luc mRNA

GTCCCGCAGTCGGCGTCCAGCGGCTCTGCTTGTTCGTGTGTGTGTCGTTGCAGGCCTT
ATTCAAGCTTGAGGATGGAAGATGCCAAGAACATCAAGAAGGGCCCTGCTCCATTC
TACCCTCTGGAAGATGGAACAGCCGGCGAGCAGCTGCACAAGGCCATGAAGAGATA
CGCTCTGGTGCCCGGCACAATCGCCTTCACAGATGCTCACATCGAGGTGGACATCAC
CTACGCCGAGTACTTCGAGATGTCTGTGCGGCTGGCCGAAGCTATGAAGCGCTACGG
CCTGAACACCAACCACAGAATCGTCGTGTGCAGCGAGAACAGCCTGCAGTTCTTCAT
GCCTGTGCTGGGCGCTCTGTTCATCGGAGTGGCTGTGGCTCCTGCCAACGACATCTA
CAACGAGCGCGAGCTGCTGAACAGCATGGGCATCTCTCAGCCCACCGTGGTGTTCGT
GTCCAAGAAGGGACTGCAGAAAATCCTGAACGTGCAGAAGAAGCTGCCCATCATCC
AGAAAATCATCATCATGGACAGCAAGACCGACTACCAGGGCTTCCAGAGCATGTAC
ACCTTCGTGACCAGCCATCTGCCACCTGGCTTCAACGAGTACGACTTCGTGCCCGAG
AGCTTCGACAGAGACAAGACAATCGCCCTGATCATGAACAGCAGCGGCTCTACCGG
ACTGCCCAAAGGTGTTGCTCTGCCTCACAGAACCGCCTGCGTCAGATTCAGCCACGC
CAGAGATCCCATCTTCGGCAACCAGATCATCCCCGACACAGCCATCCTGAGCGTGGT
GCCTTTTCACCACGGCTTCGGCATGTTCACCACACTGGGCTACCTGATCTGCGGCTTC
AGAGTGGTGCTGATGTACCGCTTCGAGGAAGAACTGTTCCTGAGAAGCCTGCAGGA
CTACAAGATCCAGTCTGCCCTGCTGGTGCCTACTCTGTTCAGCTTCTTTGCCAAGAGC
ACCCTGATCGATAAGTACGACCTGAGCAACCTGCACGAGATCGCTAGTGGCGGAGC
CCCTCTGTCTAAAGAAGTGGGCGAAGCCGTCGCCAAGAGGTTCCATCTGCCTGGCAT
CAGACAAGGCTACGGACTGACCGAGACAACCAGCGCTATCCTGATCACACCTGAGG

GCGACGATAAGCCTGGCGCTGTGGGAAAAGTGGTGCCATTCTTCGAGGCCAAGGTG
GTGGACCTGGACACCGGAAAAACACTGGGCGTTAACCAGAGGGGCGAGCTGTGTGT
CAGAGGCCCTATGATCATGAGCGGCTACGTGAACAACCCCGAGGCCACCAACGCTC
TGATCGACAAGGATGGATGGCTGCACAGCGGCGACATTGCCTACTGGGACGAAGAT
GAGCACTTCTTCATCGTGGACAGACTGAAGTCCCTGATCAAGTACAAGGGCTACCAG
GTGGCCCCTGCCGAGCTGGAATCTATCCTGCTCCAGCATCCTAACATCTTCGATGCC
GGCGTGGCAGGACTGCCTGACGATGATGCTGGCGAACTGCCTGCTGCTGTGGTGGTG
CTGGAACACGGCAAGACCATGACCGAGAAAGAAATCGTGGACTACGTGGCCAGCCA
AGTGACCACCGCCAAGAAACTGAGAGGCGGCGTGGTGTTTGTGGACGAGGTGCCAA
AAGGCCTGACCGGCAAGCTGGACGCCAGAAAGATCAGAGAGATCCTCATCAAGGCC
AAGAAAGGCGGCAAGATCGCCGTGTAGGACTAGTGCATCACATTTAAAAGCATCTC
AGCCTACCATGAGAATAAGAGAAAGAAAATGAAGATCAATAGCTTATTCATCTCTTT
TTCTTTTTCGTTGGTGTAAAGCCAACACCCTGTCTAAAAAACATAAATTTCTTTAATC
ATTTTGCCTCTTTTCTCTGTGCTTCAATTAATAAAAAATGGAAAGAACCTAGATCT

5’-UTR: 1-72
ORF (whole luciferase): 73-1725
3’-UTR: 1726-1925

Template DNA sequences for $MOG_{27\text{-}63}$-mRNA
GTCCCGCAGTCGGCGTCCAGCGGCTCTGCTTGTTCGTGTGTGTGTCGTTGCAGGCCTT
ATTCAAGCTTGAGGATGCTGGTCATGGCGCCCCGAACCGTCCTCCTGCTGCTCTCGG
CGGCCCTGGCCCTGACCGAGACCTGGGCCGGCTCCTCTCCTGGGAAAAATGCCACG
GGCATGGAGGTGGGTTGGTACCGTTCTCCCTTCTCAAGAGTGGTTCACCTCTACCGA
AATGGCAAGGACCAAGATGCAGAGCAAGCACCTATCGTGGGCATTGTTGCTGGCCT
GGCTGTCCTAGCAGTTGTGGTCATCGGAGCTGTGGTCGCTGCTGTGATGTGTAGGAG
GAAGAGTTCAGGTGGAAAAGGAGGGAGCTACTCTCAGGCTGCGTGCAGCGACAGTG
CCCAGGGCTCTGATGTGTCTCTCACAGCTTGAGACTAGTGCATCACATTTAAAAGCA
TCTCAGCCTACCATGAGAATAAGAGAAAGAAAATGAAGATCAATAGCTTATTCATC
TCTTTTTCTTTTTCGTTGGTGTAAAGCCAACACCCTGTCTAAAAAACATAAATTTCTT

TAATCATTTTGCCTCTTTTCTCTGTGCTTCAATTAATAAAAAATGGAAAGAACCTAGA
TCT

5’-UTR: 1-72

Signal peptide derived from HLA-B[41)] : 73-150

ORF (MOG_{27-63}): 151-261

Signal peptide derived from MHC class I trafficking signal[41)]: 262-429

3’-UTR: 430-629

41) Krotova K *et al.*, ***Mol Ther Oncolytics.***, 15, 166-177 (2019).

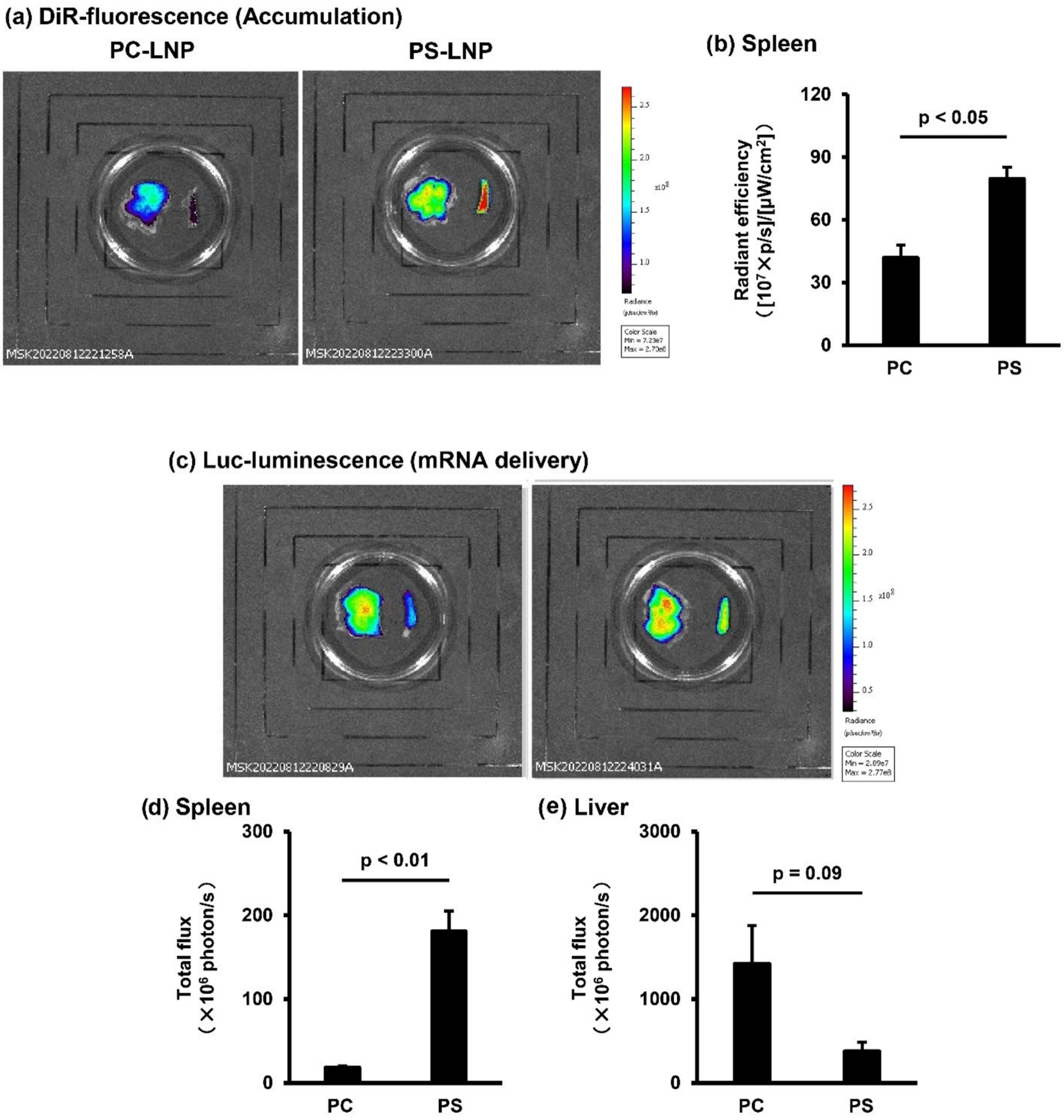

Figure S1. Biodistribution and mRNA delivery of intravenously injected LNPs. The LNPs were fluorescently labeled and loaded with luciferase mRNA. (a) The representative images of PC- and PS-LNP accumulation in the spleen and the liver. (b) Accumulation of LNPs in the spleen was evaluated through fluorescent intensity from Supplemental Figure-S1a. (c) The representative images of Luc expression by PC-LNP and PS-LNP administration. (d) mRNA delivery to the spleen was evaluated through luciferase expression from Supplemental Figure-S1c. (e) mRNA delivery to the liver was evaluated. Data represent the mean with S.E (n = 3). Student's t-test was performed between PC- and PS-LNP.

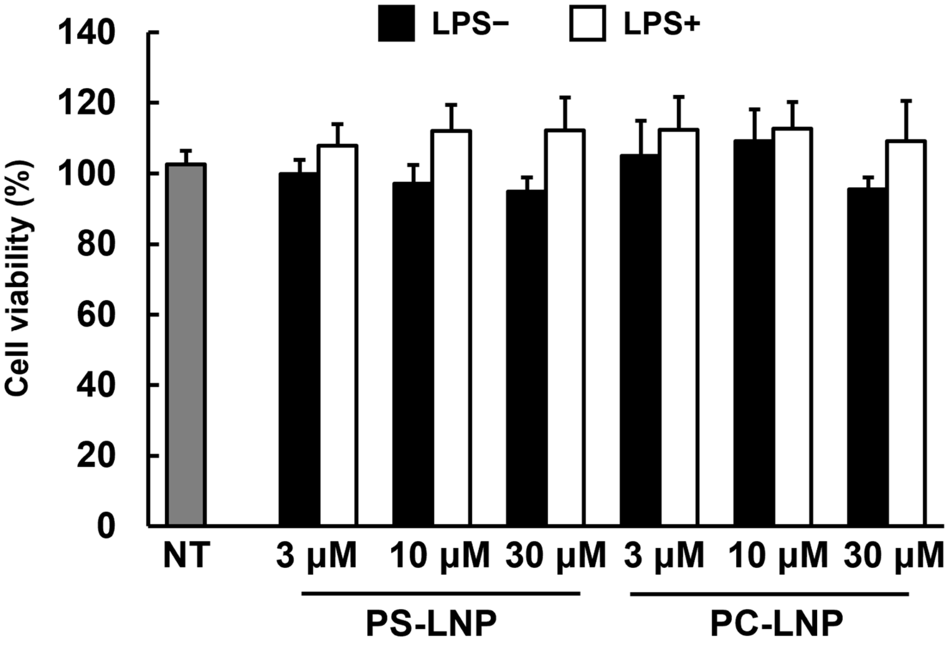

Figure S2. The effect of PS- and PC-LNP on cell viability. Raw cells were incubated with PS- or PC-LNP at indicated lipid concentration in the absence or presence of 100 ng/mL LPS at indicated lipid dosages. Cell viabilities were measured by WST-8 assay 24 hr after the addition of LNPs and LPS. Samples were set as quadruplicate.

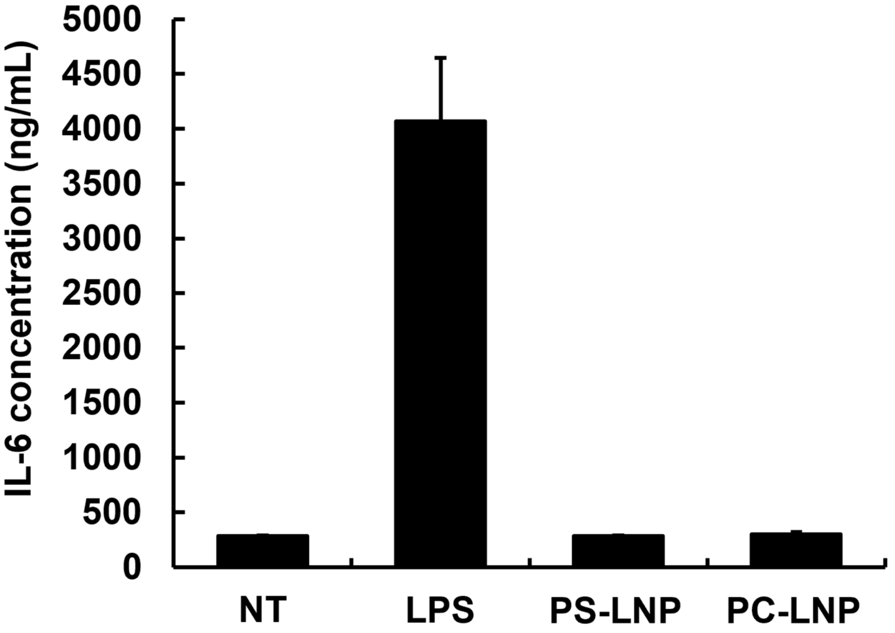

Figure S3. IL-6 production by PS- or PC-LNP in the absence of LPS. IL-6 production by PS- or PC-LNP was measured by ELISA 24 hr after the addition of LNPs at 30 μM as a lipid dosage without 100 ng/mL LPS.

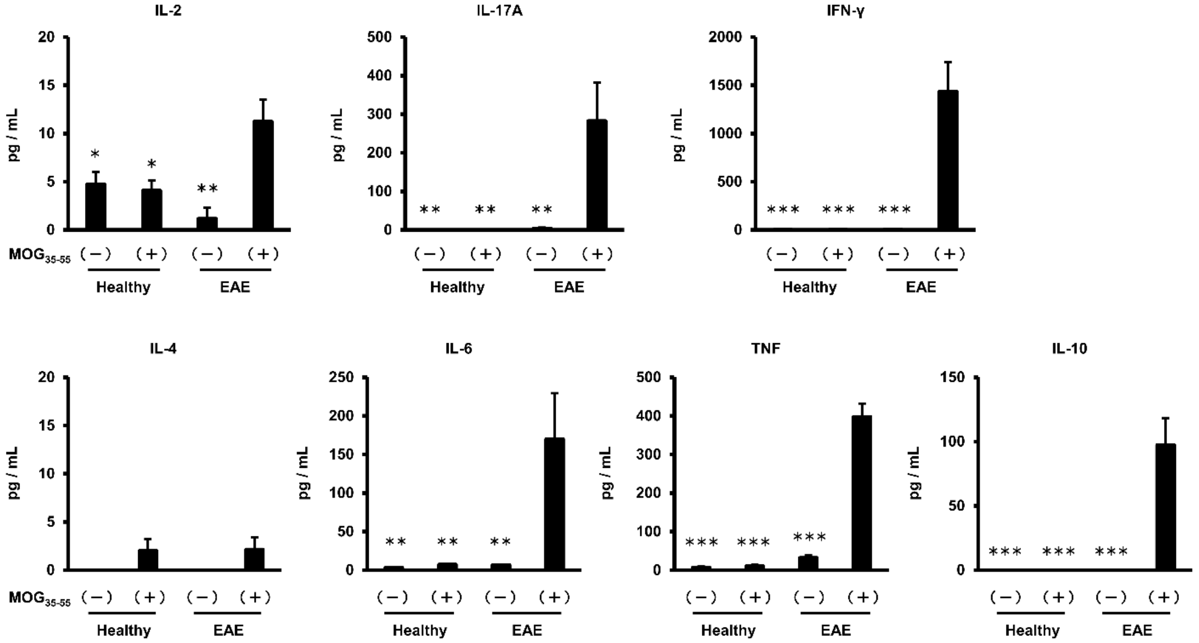

Figure S4. MOG35-55-responsive cytokine production by splenocytes. The splenocytes from EAE mice or health control mice were incubated with or without MOG35-55. Data represent the mean with S.E (n = 5). One-way ANOVA followed by Tukey's HSD test was performed. *: $p < 0.05$; **: $p < 0.01$; ***: $p < 0.001$ vs the splenocytes from EAE mice that had been restimulated with MOG35-55.

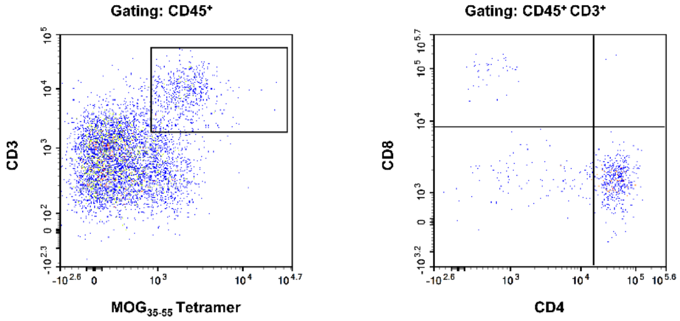

Figure S5. Gating strategy of cells from the brain. Left panel: identification of MOG_{35-55}-reactive T cells. Right panel: $CD4^+$ / $CD8^+$ separation of brain-infiltrating T cells.

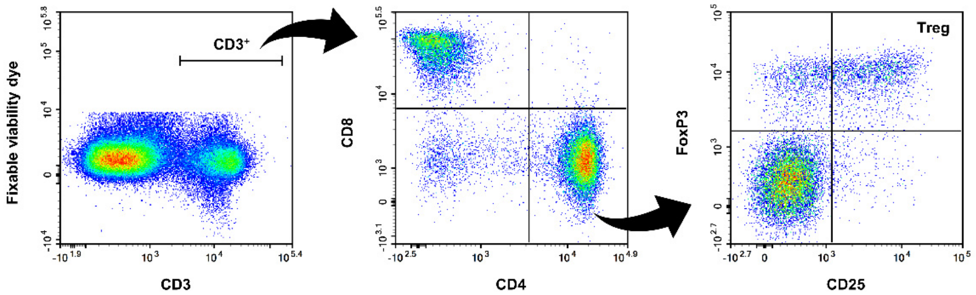

Figure S6. Gating strategy of splenocytes. Treg was identified as $CD3^{+}CD4^{+}CD25^{+}FoxP3^{+}$ (shown in the right panel).

Sample	Z-average (nm)	PdI	Zeta-potential (mV)	RR (%)	EE (%)
PC-LNP	151.4 ± 7.5	0.05 ± 0.02	-8.8 ± 1.0	76.9 ± 0.8	92.0 ± 5.4
PS-LNP	154.0 ± 3.5	0.07 ± 0.01	-24.6 ± 1.1	58.1 ± 2.8	62.0 ± 2.8

Table S1. The characteristics of the LNPs that were prepared by NanoAssemblr.

Antibody	Clone	Manufacturer	RRID
AF488 anti-CD45	30-F11	BioLegend	(BioLegend Cat# 103121, RRID:AB_493532)
APC anti-CD25	PC61	BioLegend	(BioLegend Cat# 102011, RRID:AB_312860)
BV421 anti-CD3	17A2	BioLegend	(BioLegend Cat# 100227, RRID:AB_10900227)
BV605 anti-CD4	GK1.5	BioLegend	(BioLegend Cat# 100451, RRID:AB_2564591)
PE anti-FoxP3	FJK-16s	eBioscience	(Thermo Fisher Scientific Cat# 12-5773-82, RRID:AB_465936)
PE/Cy7 anti-CD8a	53-6.7	BioLegend	(BioLegend Cat# 100721, RRID:AB_312760)
Purified anti-CD16/32	93	BioLegend	(BioLegend Cat# 101301, RRID:AB_312800)

Table S2. Antibodies used for flow cytometry.
